# Unusual Presentation of Giant Upper Limb Malignant Melanoma

**DOI:** 10.7759/cureus.16996

**Published:** 2021-08-08

**Authors:** Ooi G Khim, Juzaily F Leong, Mohamed H Sani, Nor Hazla Mohamed Haflah

**Affiliations:** 1 Orthopaedics and Traumatology, Faculty of Medicine, Universiti Kebangsaan Malaysia, Kuala Lumpur, MYS; 2 Orthopaedics, Faculty of Medicine, Unversiti Kebangsaan Malaysia, Kuala Lumpur, MYS

**Keywords:** giant melanoma, soft tissue sarcoma, metastases, adjuvant therapy, upper limb

## Abstract

Melanoma is rare among the Asian population. In Malaysia, there is little public awareness of melanoma compared to other types of cancer. Giant melanomas measuring more than 10 cm are rarely encountered and there are limited data available regarding this disease. We would like to report an unusual presentation of giant malignant melanoma of upper limb in a 62-year-old patient who presented with a five-month history of a progressively enlarging, painless mass on his left arm. This mass which measures 10 x 15 cm turned out to be a giant malignant melanoma of the left arm. To our knowledge, this is the largest melanoma of arm reported in Malaysia. Radiological findings were suggestive of soft tissue sarcoma with lung metastasis. Patient underwent wide local excision of left-arm mass and upper limb reconstruction. The diagnosis of malignant melanoma was made based on the final histopathology report. Despite aggressive treatment involving multidisciplinary units, it did not prevent the disease progression and patient succumbed five months after his presentation. Here, we present our experience in the management of this large malignant melanoma of the arm and to stress the sinister nature of melanoma.

## Introduction

Melanoma is an aggressive form of skin cancer that originates due to malignant transformation of the melanocytes. This disease is rare in the Asian population and occurs predominantly among Caucasians. The prevalence rate in Malaysia is about 0.5 per 100,000 for males and 0.3 per 100,000 for females [1]. Based on Malaysia National Cancer Registry, there were 184 and 163 reported cases of melanoma in male and female, respectively in the years 2012-2016. From these results, 58.3% male and 52.3% female were belonged to have stage IV melanoma. Giant malignant melanoma on the other hand is defined as lesions at least 10cm in diameter [2]. Del Boz et al described the largest malignant melanoma of upper limb, measuring 20 x 20 cm [3]. In Malaysia, there has only been one reported case of giant malignant melanoma of the limb. Ng BW et al reported a case of giant melanoma of the right thigh with bilateral synchronous breast carcinoma [4]. This proves malignant melanoma; especially giant malignant melanoma is rare and carries a high morbidity and mortality rate which is attributed to the late detection of the disease. 

## Case presentation

**Figure 1:**
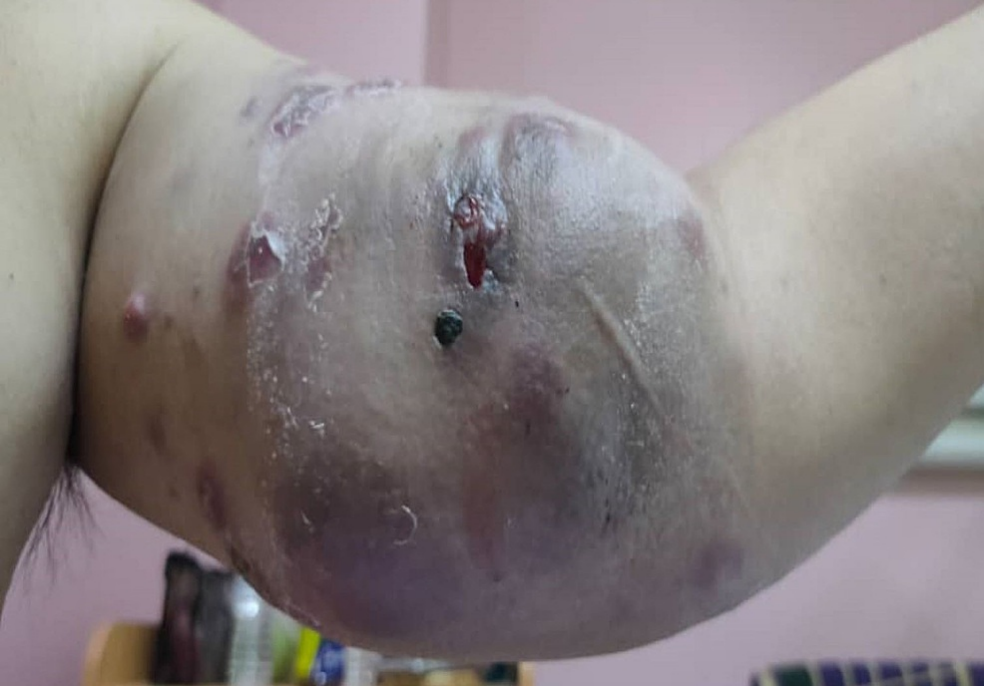
Large mass on anteromedial aspect of left arm with areas of pigmentation.

A 62-year-old man with a background history of diabetes mellitus presented with a five-month history of progressively enlarging, painless mass on the anteromedial aspect his left arm (Figure 1). It was associated with constitutional symptoms, whereby patient claims to have lost 9 kg in the period of three months. He also noticed several areas of pigmented lesion over the mass which were painless. Over time, the swelling started to ulcerate with surrounding rashes, which prompted the patient to seek medical attention. There was no history of malignancy in his family. His occupation as an engineer in the Middle East caused him to have prolonged exposure to ultraviolet light. On examination, there was a large mass located on the anteromedial aspect of his left arm. The overlying skin has a bluish discoloration with several areas of pigmentation and presence of dilated veins. Firm in consistency with an ill-defined margin, it was adherent to the overlying skin and to the underlying muscle. No palpable thrill was detected to suggest a vascular in origin. Tinel test was also negative excluding that lesion arises from a nerve. A small subcutaneous nodule was located proximally to the lesion. Despite the size of the lesion, there was no limitation of movement at the elbow or shoulder. Distally the neurovascular bundle was not compromised. Proximally, a smaller mass was detected in the axilla which was suspicious of an enlarged lymph node. The presence of an enlarged lymph nodes led us to surmise that lymphoma was a more likely diagnosis than a soft tissue sarcoma.

**Figure 2:**
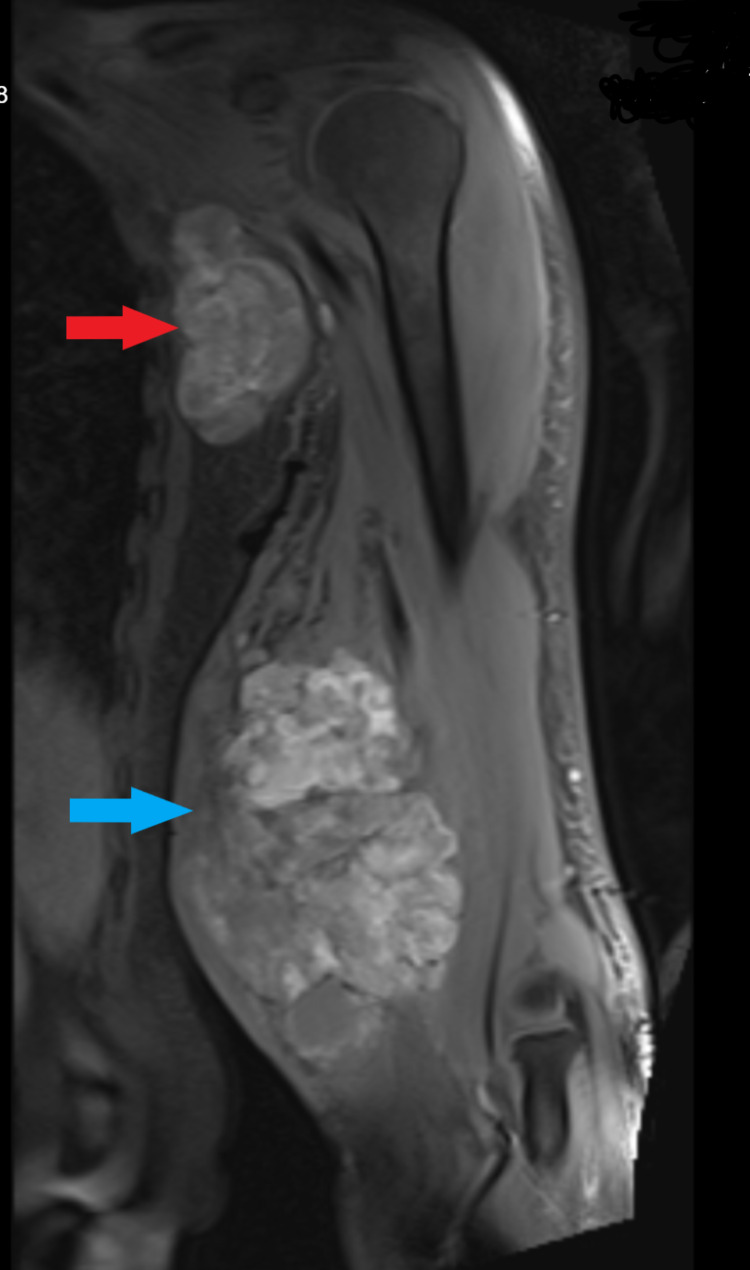
MRI of the arm showed heterogenous mass on the medial aspect of the left arm (blue arrow) and matted axillary lymph nodes (red arrow).

**Figure 3:**
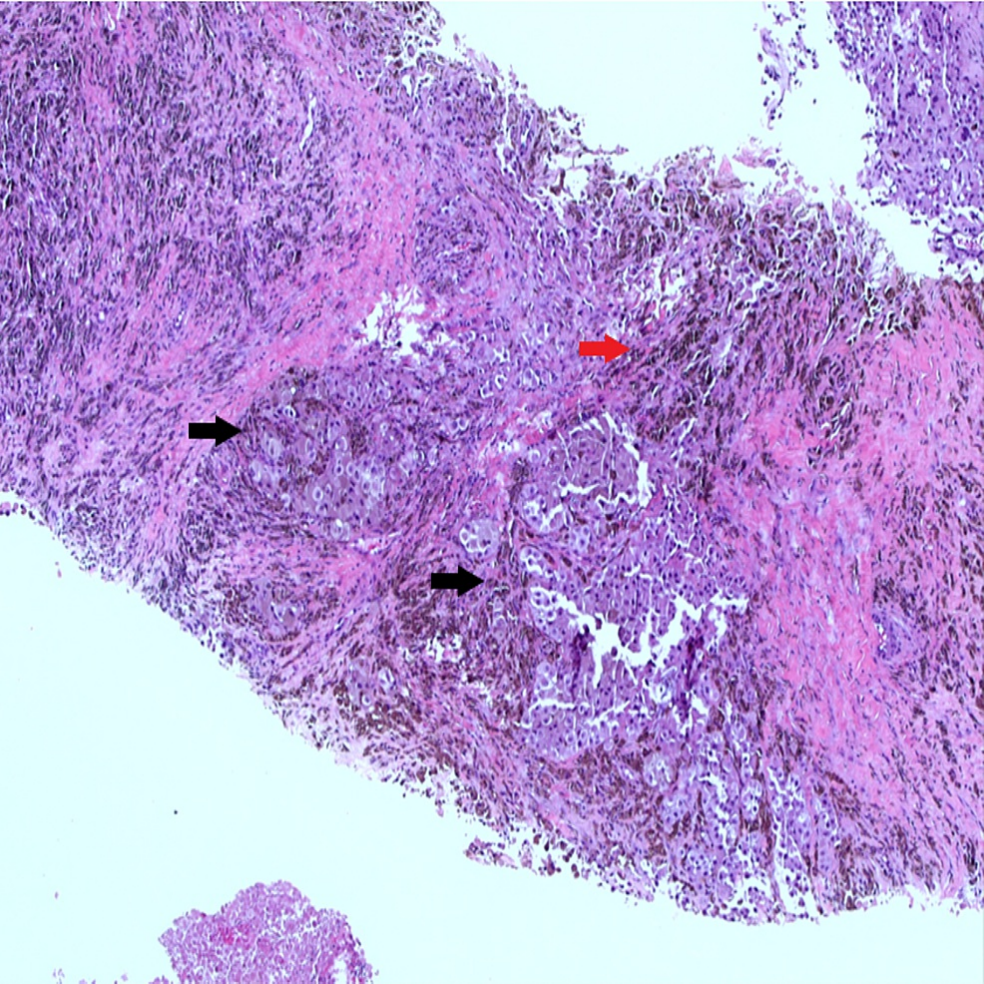
: Microscopic examination shows infiltration by malignant cells arranged in sheets (black arrow) and the surrounding stroma shows dense melanin pigment deposition (red arrow).

With the above differential diagnosis, staging was next step in the management of this patient. Magnetic resonance imaging (MRI) illustrated a large heterogenous multilobulated left arm mass (Figure 2). The fascia is breached, involving the subcutaneous layer and skin with presence of satellite nodules surrounding the mass. The neurovascular bundles were displaced laterally, however, displayed normal flow signal. There are enlarged matted left axillary lymph nodes, with the presence of necrotic center. Tru-cut biopsy was performed, and histopathology revealed malignant melanoma (Figure 3) as evidenced by infiltration by malignant cells arranged in sheets and the surrounding stroma shows dense melanin pigment deposition. Subsequently, PET scan was performed as part of staging for malignant melanoma which reported a hypermetabolic multilobulated soft tissue mass at medial aspect of left arm likely primary lesion with nodal and lung metastases.

**Figure 4:**
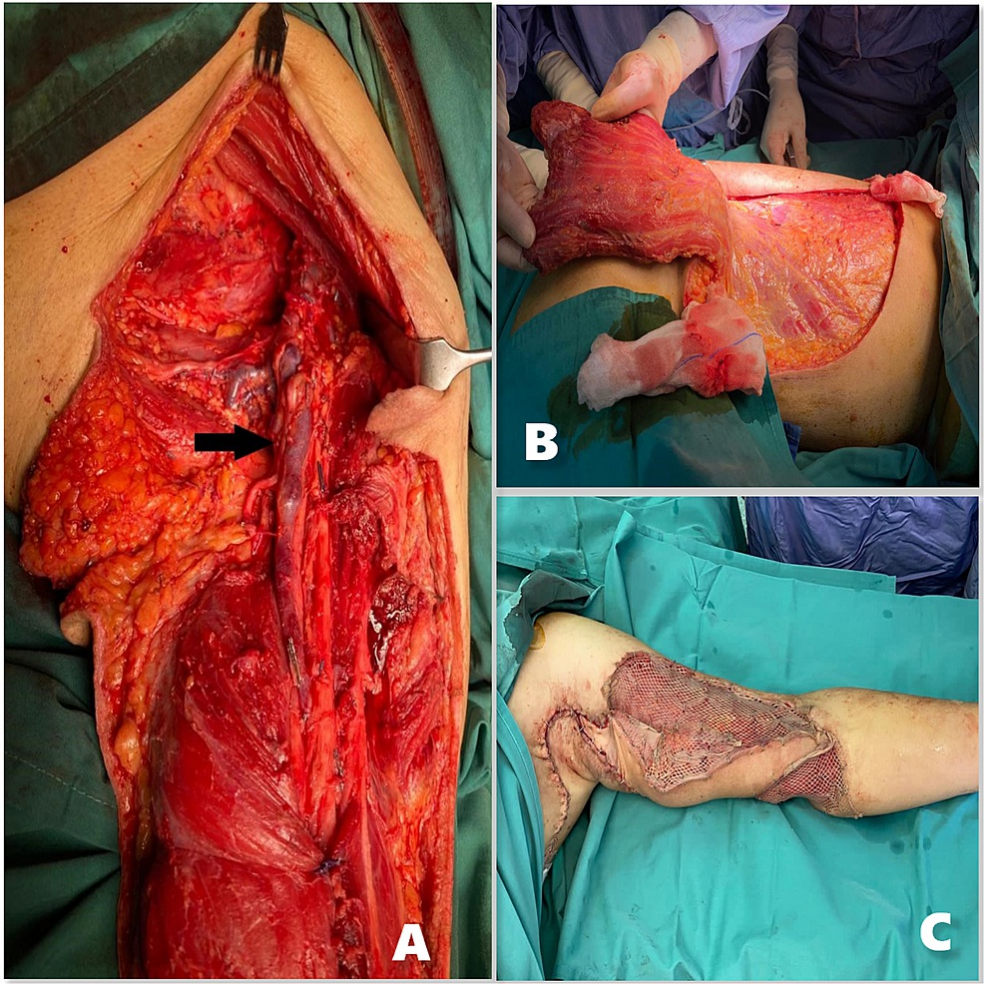
A: Exposed neurovascular structures (black arrow) with large skin defect. B: Latissimus dorsi flap. C: Left arm reconstruction with functional latissimus dorsi flap and split skin graft from the left thigh.

**Figure 5:**
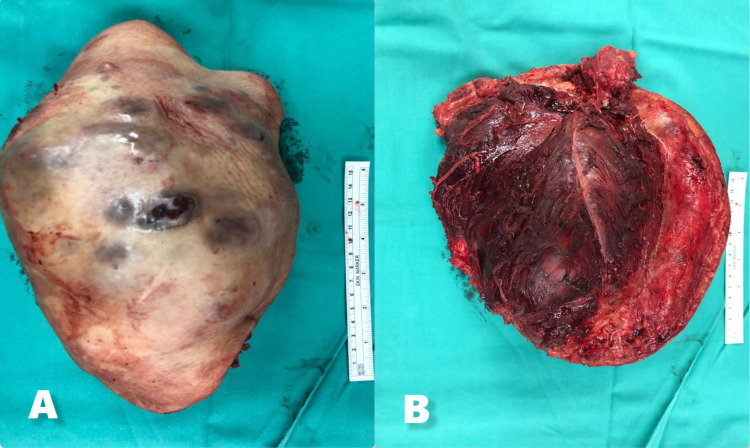
A: Left arm mass 9 x 10 x 15 cm (anterior). B: Left arm mass with biceps muscle (posterior).

**Figure 6:**
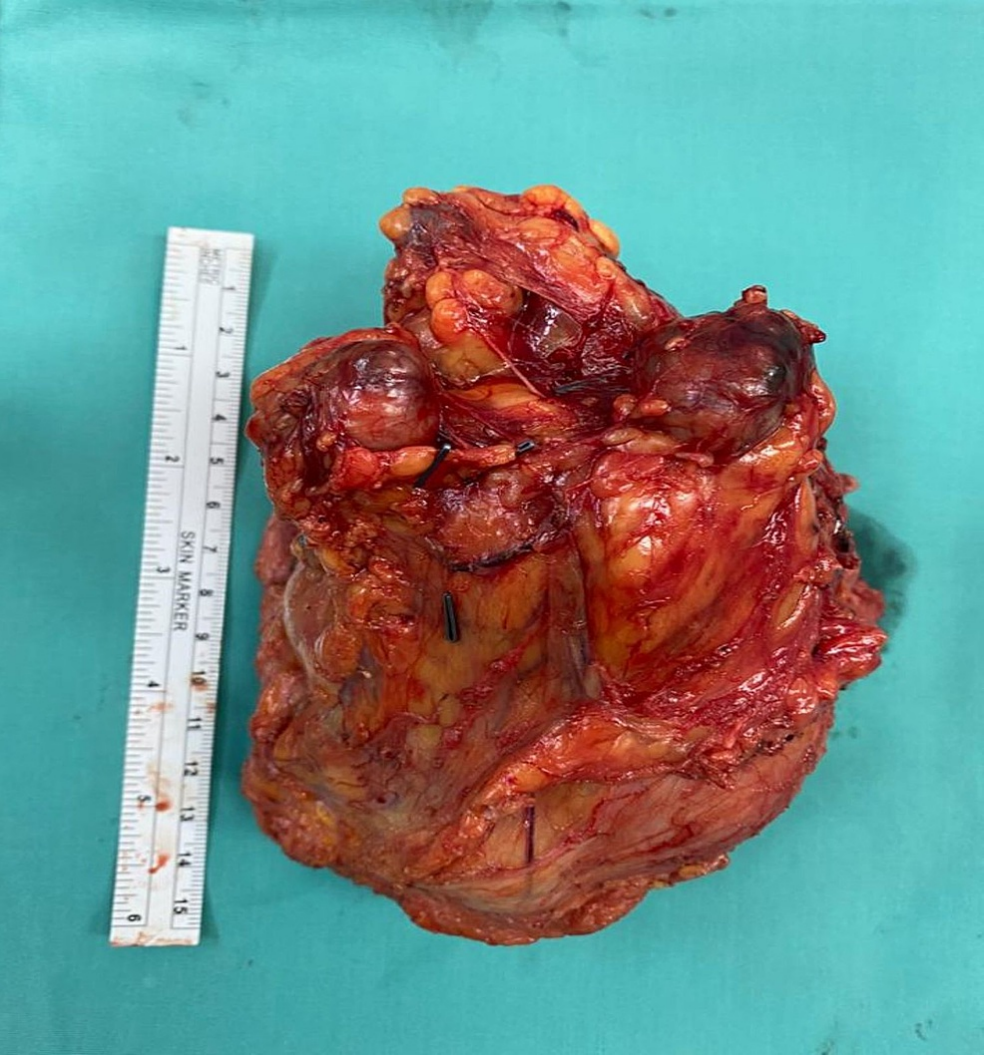
Left axillary mass.

Patient underwent wide local excision, axillary dissection of the left arm malignant melanoma followed by left upper limb reconstruction with left functional latissimus dorsi flap with split skin graft harvested from the left thigh (Figure 4). Intraoperatively, the left arm mass measured 9 x 10 x 15 cm (Figure 5) and was adherent to the underlying biceps muscle. There were several subcutaneous nodules surrounding the mass that was only detected by palpation. The neurovascular bundles although closely related to the tumor was not encased in it. The axillary mass measured 8 x 5 x 6 cm (Figure 6) was not connected to left arm mass. Postoperatively, patient recovered well with no neurological deficit.

Final histology showed a pT4bN3 malignant melanoma with a Clark’s level of V and Breslow tumor thickness of >10 mm with lymphovascular invasion. Further histological testing showed the tumor to be strongly positive for S100 and Melan-A BRAF mutation was also detected in this patient, which directed our oncologist towards commencement of dabrafenib and trametinib for this patient. Unfortunately, patient succumbed to brain metastasis prior to treatment.

## Discussion

The incidence and mortality rate of malignant melanoma are rising over the last decade due to its aggressive nature. This disease commonly affects the white population and equal on both genders [5]. The etiology remains unclear. However, several risk factors have been recognized, which include ultraviolet radiation from sun exposure, genetic factors, lighter-skinned phenotype and pre-existing giant congenital melanocytic nevi (GCMN) [6].

At initial presentation, a preliminary diagnosis of soft tissue sarcoma was made. A firm, large soft tissue mass (> 5cm) which is deep (in relation to investing fascia) should raise suspicion of malignant soft tissue tumor [7]. Ulceration and bleeding from a soft tissue sarcoma is not unusual due to blood supply compromise in an enlarging tumor. Additionally, the medial aspect of the upper limb which is a region that is not exposed to the sun is a rare site for malignant melanoma. However, there were several clues that would cause one to suspect that this lesion was a skin in origin. Firstly, the patient gave a history of the appearance of pigmented lesions surrounding the lump which was apparent on clinical examination. Although he was unable to determine accurately the timeline, whether it appeared before or after the swelling. Furthermore, the tumor was adherent to the skin which should raise the suspicion that the tumor is skin in origin. An occupational history involving prolonged sun exposure was a possible risk factor in this case.

Due to the rare nature of this tumor, data on giant malignant melanoma is mostly based on case reports. Gishen et al found 19 reported cases of giant malignant melanoma between 1960 to 2016 [8]. The most common location was in the trunk followed by the extremities. Of 19, three were located in the arm with largest size being 20 x 15 x 7 cm. This tumor is highly associated with regional nodal or systemic metastasis with only four reported cases of a localized disease. Metastasis, tumor depth and thickness and ulceration were prognostic determinant [6,8].

Recommended treatment for giant malignant melanoma with Breslow depth of 4 mm or greater is wide local excision with minimum excision margin of 2cm [9]. Death in giant malignant melanoma with a thickness of 4 mm is most likely secondary to metastasis than local recurrence, thus negating the benefit of an excision margin of more than 2 cm [9]. In case of regional lymph node metastasis, radical dissection is a standard therapy however was not found to improve the overall survival rate [6].

Radiation therapy should be considered in the case of giant melanoma despite it being a radioresistant tumour [7]. It has a role as primary treatment in case of inoperable or recurrent tumor and provides palliative treatment in case of advanced stage and metastatic disease. Radiotherapy also may be used as adjuvant therapy after complete excision of the tumor or after lymphadenectomy to improve local and regional control. However, there is limited data to prove radiotherapy can benefit on overall survival rates [8]. 

There has been advancement with regards to adjuvant therapy in advanced malignant melanoma. The usage of interferon (IFN) alfa in the treatment of advanced-stage melanoma has declined over the past years due to the risk of toxicity and lack of clear survival benefits [10]. Based on recent meta-analyses of trials, interferon alfa revealed a significant effect on relapsed free survival, but only 3% overall survival rates [6,10]. Studies on immunotherapy using checkpoint inhibitors against cytotoxic T-lymphocyte-associated protein-4 (CTLA4) such as ipilimumab have shown encouraging results [11]. BRAF gene mutation is present in approximately half of the advanced melanomas with V600E being the most common mutation [12]. Trials have shown good response with BRAF and/or MEK inhibitors therapy [6]. Dacarbazine which is the most established chemotherapy used can be considered when immunotherapy or targeted therapy is not available [13]. The introduction of combination BRAF and MEK inhibitor therapy has transformed treatment outcomes in patients with advanced-stage BRAF-mutated melanoma [12]. A five-year overall survival rates for metastatic melanoma have increased substantially from less than 10% to up to 40%-50% [6].

## Conclusions

In conclusion, the incidence of giant malignant melanoma is rare. It poses a great surgical challenge due to its size and destructive nature. We would like to highlight the sinister nature of this disease, which warrants an early detection for a better prognosis. Public awareness plays a crucial role in the early detection of the disease, as our case, the patient took five months before seeking medical attention. The late presentation with distant metastasis resulted in a guarded prognosis.
